# Molecular logic of salt taste reception in special reference to transmembrane channel-like 4 (TMC4)

**DOI:** 10.1186/s12576-022-00856-y

**Published:** 2022-11-30

**Authors:** Yoichi Kasahara, Masataka Narukawa, Ayako Takeuchi, Makoto Tominaga, Keiko Abe, Tomiko Asakura

**Affiliations:** 1 Department of Applied Biological Chemistry, Graduate School of Agricultural and Life Sciences, The University of Tokyo, 1-1-1 Yayoi, Bunkyo-Ku, Tokyo, 113-8657 Japan; 2 Department of Food and Nutrition, Kyoto Women’s University, 35 Kitahiyoshicho Imakumano, Higashiyama, Kyoto, 605-8501 Japan; 3 Department of Integrative and Systems Physiology, Faculty of Medical Sciences, and Life Science Innovation Center, University of Fukui, Fukui, 910-1193 Japan; 4 Division of Cell Signaling, National Institute for Physiological Sciences, National Institutes of Natural Sciences, 5-1 Aza-Higashiyama, Myodaijicho, Okazaki, Aichi 444-8787 Japan; 5 Thermal Biology Research Group, Exploratory Research Center on Life and Living Systems (ExCELLS), National Institutes of Natural Sciences, 5-1 Aza-Higashiyama, Myodaijicho, Okazaki, Aichi 444-8787 Japan; 6 Kanagawa Institute of Industrial Science and Technology (KISTEC), Life Science & Environment Research Center (LiSE), 3-25-13 Tonomachi Kawasaki-Ku, Kawasaki, Kanagawa 210-0821 Japan

**Keywords:** Salty taste, TMC4, Chloride channel, Anion effect, Amiloride-insensitive

## Abstract

The taste is biologically of intrinsic importance. It almost momentarily perceives environmental stimuli for better survival. In the early 2000s, research into taste reception was greatly developed with discovery of the receptors. However, the mechanism of salt taste reception is not fully elucidated yet and many questions still remain. At present, next-generation sequencing and genome-editing technologies are available which would become pivotal tools to elucidate the remaining issues. Here we review current mechanisms of salt taste reception in particular and characterize the properties of transmembrane channel-like 4 as a novel salt taste-related molecule that we found using these sophisticated tools.

## Background

Salt is the oldest and most commonly recognized seasoning. The most representative salt is sodium chloride, which has an authentic salty taste. In Romania, the ruins of a salt-making facility, built approximately 8000 years ago, were discovered. Even long before salt was already used as a seasoning by the time humans began cooking with fire [1]. In addition to its use as a seasoning, salt is an indispensable ingredient for food preservation and processing. In recent years, however, it has been reported that excessive salt intake increases the risk of various physiological disorders, e.g., vascular and renal dysfunctions [2]. Therefore, in 2012, the World Health Organization (WHO) strongly recommended that the daily salt intake for adults in general be set at 5 g/day [3]. In each country, various measures are being taken to reduce salt under the government’s initiative, but few countries are able to achieve the WHO’s goals [4]. To reduce the incidence of excessive salt intake, it is important to understand the underlying mechanism of salt taste reception. If the molecular mechanism of salt taste reception is clarified, it may be possible to efficiently develop a salt taste enhancer that strengthens the salty taste with even with a small amount of Na^+^. This review overviews previous reports and recent findings on the reception of salty taste, as well as the properties of novel voltage-dependent chloride channels, such as transmembrane channel-like 4 (TMC4).

### Mammalian taste reception

**Fig. 1:**
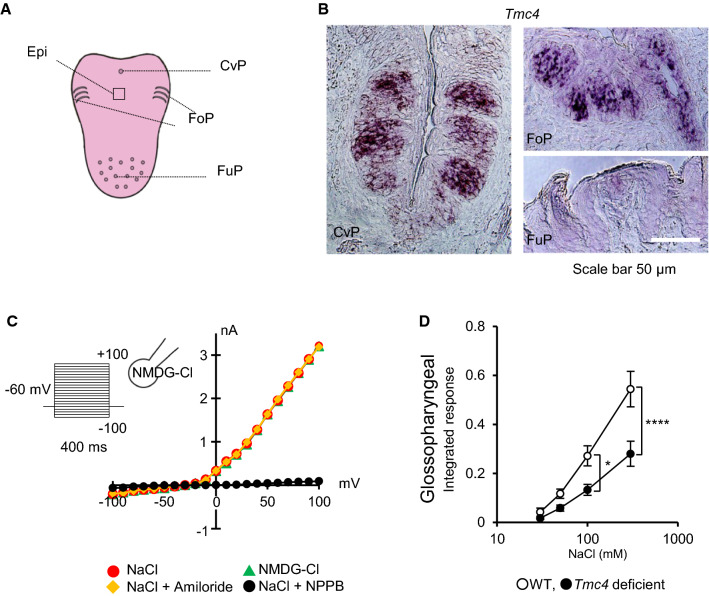
TMC4 is a novel voltage-dependent chloride channel that is specifically expressed in the posterior part of the tongue and is responsible for the salt response of the glossopharyngeal nerve. **A** Location of the circumvallate papillae (CvP), foliate papillae (FoP), and fungiform papillae (FuP) on a mouse tongue. Surrounding epithelia (Epi) means to the epithelia of the tongue that does not contain taste papillae. **B** In situ hybridization of *Tmc4* mRNA is specifically detected in the CvP and FoP, but barely detected in the FuP. **C** Representative I-V relationship of the currents using step pulses (the holding potential was -60 mV, and stimulated from − 100 to + 100 mV [∆10 mV] for 400 ms) under different bath solutions. The mouse TMC4-mediated outward current is not affected by the bath application of *N*-methyl-D-glucamine (NMDG)-Cl, NaCl, or amiloride (epithelial sodium channel inhibitor), but is significantly reduced by the anion channel inhibitor, 5-nitro-2-(3-phenylpropylamino) benzoic acid (NPPB). D) Integral glossopharyngeal nerve response of wild-type (WT) or *Tmc4*-deficient mice to NaCl solutions (30, 50, 100, and 300 mM). The asterisks (^*^, ^****^) indicate statistical differences: *p* < 0.05 and *p* < 0.0001, respectively. Significance was evaluated using two-way ANOVA with Bonferroni correction (*n* = 6–7 for WT and *n* = 5–6 for KO)

In mammals, taste buds receive taste stimuli from the oral cavity. Approximately two-thirds of all taste buds are located in the papillae tissues of the tongue (fungiform, foliate, and circumvallate papillae; Fig. 1A). The remaining one-third of the taste buds are located in the pharynx and epithelium of the soft palate [5, 6]. The fungiform papillae on the anterior part of the tongue mainly project onto the chorda tympani nerve, and the circumvallate and foliate papillae on the posterior part of the tongue project onto the glossopharyngeal nerve. The taste buds of the pharynx and throat are thought to be involved in the swallowing reflex because they have a more powerful response to water stimuli than taste stimuli [7]. One unit of taste buds is a bud-shaped tissue with a diameter of approximately 50 μm and a height of approximately 80 μm, which comprises approximately 50–150 taste cells [6]. The tip of the taste bud is located in the surface of the tongue and has a small opening, called the taste pore. Parts of the food dissolved in saliva come into contact with taste cells through the pore.

Based on their morphological characteristics, taste cells are classified into types I to IV [8]. Type II cells are involved in sweet, umami, and bitter tastes, whereas type III cells are involved in sour taste [5, 9]. Type III cells are the only taste cells in which neural connections are observed. Blocking these neural connections reduced sour taste responses [9].

Taste receptors related to the five basic tastes are expressed in the taste cells. These taste receptors are thought to function in taste pore located on the apical membrane of taste cells. Specific G protein-coupled receptors are believed to accept sweet, umami, and bitter tastes, whereas ion channels accept sour and salty tastes [5]. In the early 2000s, research on taste receptors evolved dramatically, with receptors for sweet, umami, and bitter taste being identified [5]. As for the sour taste receptor, various candidate molecules have been reported. Of these, Otopetrin1, which was reported in 2018, is presently the most promising molecule [10]. However, salt taste receptors have not yet been thoroughly explored.

### Two salt taste components with different responses to amiloride

Salt taste reception is divided into two components, amiloride-sensitive and amiloride-insensitive reception, based on the difference in responsiveness to amiloride, which is an inhibitor of the epithelial Na^+^ channel (ENaC) [11]. Amiloride-sensitive salt taste is received by fungiform papillae in the anterior part of the tongue, which is projected onto the chorda tympani nerve [12, 13]. In contrast, amiloride-insensitive salt taste is received by circumvallate and foliate papillae in the posterior part of the tongue, which is projected onto the glossopharyngeal nerve [14, 15]. The glossopharyngeal nerves are less affected by amiloride [14]. In the taste cells of mice, the subunits of ENaCα, ENaCβ, and ENaCγ are thought to form and function as heterotrimeric ENaCαβγ [16]. Similarly in other tissues, including the kidney, lung, distal colon, and sweat duct, ENaC also functions in the heterotrimer, ENaCαβγ [17–19]. In the heterologous expression system using *Xenopus* oocytes, expression of one or two ENaC subunits results in reduced or no detectable amiloride-sensitive Na^+^ current [20]. The ENaCδ subunits are also expressed in human tissues, such as brain and function in ENaCδβγ, but there are no orthologs of ENaCδ in mice or rats [21, 22].

Mice generally prefer approximately 100 mM NaCl and aversive high concentrations of NaCl (above 300 mM). In ENaCα knockout (KO) mice, the preference for salt response is reduced; therefore, ENaCαβγ is thought to be responsible for preference (low-concentration, amiloride-sensitive) salt taste reception [15]. In contrast, high-concentration salts are considered to be aversive salts (amiloride-insensitive), but their specific receptor is unknown.

### Localization of ENaCαβγ in mouse taste buds

Nomura et al. reported that approximately half of ENaCα in fungiform papillae is co-expressed with calcium homeostasis modulator 3(CALHM3), and the ENaCα mediates amiloride-sensitive salt taste reception, through the CALHM1/CALHM3 channel [23]. CALHM1/CALHM3 was recently identified as an ATP release channel in type II cells [24]. Supporting this, Ohmoto et al*.* reported that the *Skn-1a* gene, which is a transcription factor of type II cells, is also involved in amiloride-sensitive salt taste reception using *Skn-1a* KO mice and *Calhm3* KO mice [25]. These results suggest that at least a part of ENaCα in the fungiform papilla is expressed in type II cells. On the other hand, a recent study using transgenic mice with fluorescently labeled ENaCα and β has reported that each monomer ENaC was not distinctly co-expressed in taste cells. This indicates that ENaC may not form trimers in taste cells [26]. Therefore, it may not function as ENaCαβγ in taste cells. This report showed that ENaCα is expressed in the fungiform and circumvallate papillae of type III cells, whereas ENaCβ is expressed in the fungiform papillae of type I cells. The expression of ENaCγ was confirmed in type II cells in approximately 70% of the fungiform papillae [26].

It is an undeniable fact that amiloride has a great influence on salt taste reception in mice, and the molecule strongly related to the reception of amiloride is ENaC. However, further studies are needed to determine the stoichiometry of ENaC subunits, and how ENaC functions in taste cells.

### Effects of ENaC and amiloride on human salt taste reception

Using immunohistochemical staining, Stähler et al. found that ENaCβ, ENaCγ, and ENaCδ subunits are expressed in human fungiform and circumvallate papillae [27]. They also reported that ENaCα was expressed only in the taste cells of the circumvallate papilla, and only ENaCδ was expressed in the taste pores of the taste buds. Thus, the trimer of ENaCαβγ or ENaCδβγ was considered to work in human taste buds. From cellular experiments, the 50% inhibition concentration (IC_50_) of amiloride for ENaCδβγ (IC_50_ ≒ 2.7 μM) is about 25 times higher than that for ENaCαβγ (IC_50_ ≒ 0.11 μM) [28, 29]. It is not known whether this is the direct cause, but the salt receptivity in human is less affected by amiloride than in mouse [30, 31]. Therefore, we suggest that amiloride-sensitive salt taste reception through ENaC does not significantly affect salt taste reception in humans. Human salt taste reception may involve the amiloride-insensitive salt taste pathway; thus, it is important to clarify amiloride-insensitive salt taste reception to understand human salt taste reception.

### Amiloride-insensitive salt taste reception

TRPV1t, a taste variant of the transient receptor potential vanilloid 1(TRPV1), has been reported as a candidate of amiloride-insensitive salt receptor [32]. TRPV1 is a well-known voltage-dependent cation channel involved in the reception of temperature and pain in the nerve cells [33]. However, other reports using TRPV1 KO mice have not presented clear data implicating TRPV1 in amiloride-insensitive salt taste reception [34–36]. In addition, TRPV1 is barely expressed in isolated taste cells [37].

It is also believed that type II and III taste cells are involved in the amiloride-insensitive salt response. *Transient receptor potential cation channel subfamily melastatin 5* (*Trpm5*) is a molecule required for sweet, umami, and bitter taste signaling expressed in type II cells [38]. *Polycystic kidney disease 2-like 1* (*Pkd2l1*) is a marker of type III cells. *Pkd2l1*-TeNT shows mice in which nerves connecting to type III cells were blocked using diphtheria toxin and *Pkd2l1*-positive cells [9]. *Trpm5* KO/*Pkd2l1*-TeNT double mutant mice had significantly reduced amiloride-insensitive salt responses compared to WT [39]. In contrast, each single-mutant, *Trpm5* KO and *Pkd2l1*-TeNT mice are not significantly different from WT in salt taste responses [9, 38]. However, the specific receptor molecule involved in amiloride-insensitive salt taste reception remains unclear.

### Anion effect of salt taste

It is important to note that NaCl is not the only molecule with a salty taste, as KCl also has a salty taste. For this reason, most researchers believe that the mechanism behind cation reception is the key to elucidating the mechanism of salt taste reception. However, Na salts in which the ‘‘Cl^−^’’ ion is replaced with other anions (e.g. Na_2_SO_4_, NaOCOCH_3_, NaHCO_3_) have less of a salty taste than those with the same concentration of NaCl [40]. The salts with a higher molecular weight of Na salt, such as Na-gluconate, also have less of a salty taste. Ye et al. called this phenomenon the “anion paradox” or “anion effect,” and attributed it to the large molecular size of anions that inhibit the depolarization of taste cells by Na^+^ [41]. Elliott et al*.* reported that in rats, the response of the chorda tympani nerves to NaCl was not suppressed by bumetanide, an inhibitor of Na^+^-K^+^-2Cl^–^ cotransporter (NKCC) and neither was it suppressed by 4-acetamido-4′-isothiocyanostilbene-2,2′-disulfonic acid, 4,4′-diisothiocyanato-2,2′- stilbenedisulfonic acid (DIDS), or 9-anthracene carboxylic acid, which are inhibitors of chloride channels [42]. As a result, until recent years, it was thought that taste cells that are involved in salt taste do not have Cl^−^ receptor molecules, and Cl^−^ flows through tight junctions between taste cells. Research on the role of Cl^−^ in salt taste reception is scarce, and no major research progress has been reported since the beginning of 2000. Using the Ca^2+^ imaging method, Lewandowski et al*.* reported that isolated type III taste cells from circumvallate papillae in mice responded differently, depending on the type of anion [43]. Roebber et al*.* also showed that isolated taste cells of the fungiform papillae in mice have anion sensitivity for salt taste reception [44]. These two reports greatly updated the conventional anion hypothesis, and suggested that taste cells have Cl^−^ receptors for salt taste reception. However, a specific anion channel responsible for salty taste has not yet been identified. Genetic analysis of isolated taste cells has reported that some anion channels, e.g., *Ano1, Ano10, cystic fibrosis transmembrane conductance regulator,* are expressed in taste cells, but it is not clear how these anion channels are involved in taste responses [37].

### Discovery of TMC4, a molecule that is specifically expressed in taste cells

Based on the above-mentioned background, the salt taste receptor of human is unknown, and the only clue to finding this receptor is that human salt taste reception is unaffected by amiloride. To elucidate the amiloride-insensitive mechanism, we focused on molecules specifically expressed in the epithelium of circumvallate papillae. The circumvallate papillae comprise a tissue of the tongue that projects onto the glossopharyngeal nerve, which is less affected by amiloride. Total RNA was extracted from the epithelium of the circumvallate papilla and the surrounding tissue, and the mRNA of each tissue was compared using next-generation sequencing analysis. Each expression level was normalized based on the reads per kilobase of exon per million mapped reads (RPKM) value, and the RPKM value was used. From approximately 20,000 genes, 1120 candidate genes were identified from the threshold (RPKM value; CvP > 10, the ratio of RPKM value; CvP/Epi > 3) set considering the specific expression of taste cell markers. We focused on the TMC family, which, in mammals, consists of eight members (TMC1–TMC8). This is because *C. elegans* TMC1 has been reported as a sodium-sensitive membrane protein [45]. Among the TMC family, only *Tmc4* was included in the candidate genes, and its RPKM value was the same as that of typical taste cell markers, such as *Entpd2, Plcβ2,* and *Pkd1l3*. Staining images of the tongue tissue using in situ hybridization revealed that *Tmc4* was specifically and strongly expressed in the circumvallate papillae and foliate papillae of the posterior part of the tongue (Fig. 1B). In contrast, *Tmc4* expression in the fungiform papillae was very weak (Fig. 1B). Double staining with several taste cell markers also showed that *Tmc4* was widely expressed in taste cells of the circumvallate papillae [46].

### Function analysis of TMC4

To elucidate the properties of the membrane protein TMC4, whose function is unknown, we analyzed its function using the whole-cell patch-clamp method. When human embryonic kidney (HEK) 293 T cells expressing mouse TMC4 (mTMC4) were stimulated with step pulses, a large outward current was observed under a positive potential when compared with that of the mock cells. mTMC4-mediated currents were unaffected by intracellular and extracellular K^+^, Na^+^. These currents were independent of the addition of amiloride, but were completely diminished by the addition of the anion channel inhibitor, 5-nitro-2-(3-phenylpropylamino) benzoic acid (NPPB) [47] (Fig. 1C). mTMC4-mediated currents were also affected by several chloride channel inhibitors, such as calcium-activated chloride channel inhibitor, CaCC(inh)-A01 [48] and fluoxetine hydrochloride [49]; however, niflumic acid [50] and DIDS [51] hardly affected them.

To confirm whether mTMC4 exhibits anion permeability in a concentration-dependent manner, the Cl^−^ contained in the bath solution was gradually replaced with gluconate ions, which are large ions difficult to permeate through ion channels. Consequently, the outward current of mTMC4 decreased as the Cl^−^ concentration in the bath solution decreased. However, even when all the Cl^−^ contained in the bath solution was replaced with gluconate ions, the outward current did not completely disappear. Similarly, this outward current was retained even when all the Cl^−^ ions were replaced with aspartate and glutamate ions. These results suggest that TMC4 is a novel voltage-dependent chloride channel that allows anions to pass passively from outside the cell to within the cell, according to the anion concentration, under a positive potential [46]. Furthermore, it was suggested that TMC4 has relatively large pores [46]. Using the same method, we confirmed that human TMC4 (hTMC4) has the same properties as mTMC4.

### The molecular properties of TMC4 compared with those of other TMC families

TMC4 is the first discovered anion channel in a molecule belonging to the TMC family [40, 46]. The TMC family has a relatively conserved sequence called the “TMC domain.” This family contains 8 or 10 transmembrane domains [52–54]. With the exception of TMC1 and TMC2, the molecular properties of the TMC family are unknown. The most advanced studies of the mammalian TMC family are on TMC1, which is expressed in the hair cells of the inner ears of mice, and is closely associated with the cause of deafness upon receiving vibrational stimuli [55]. In addition, using green turtle (CmTMC1) and budgerigar (MuTMC2) analyses, TMC1 and TMC2 have recently been reported to be mechano-sensing cation channels [56]. Interestingly, neither the three amino acids G520, M521, and D672, which are important residues for cation channel functions in the structural analysis of the TMC1 and TMC2, are conserved in TMC4 [56].

Reports on other TMC molecules are limited to those in the field of gene expression analysis. Mutations in TMC6 (EVER1) and TMC8 (EVER2) may be involved in epidermodysplasia of the pancreas, also known as Tree Man syndrome [57, 58], and increased expression of *Tmc5* may be involved in the proliferation of prostate cancer [59]. Specifically, it has been suggested that the expression of *Tmc7* is enhanced at the onset of pancreatic cancer; therefore, it is expected to be a biomarker at the onset of pancreatic cancer [60]. Thus, most genes belonging to the TMC family play important physiological roles.

### The molecular properties of TMC4 compared with those of the anoctamin families

The TMC family is also classified as an anoctamin superfamily (ANO1 is also known as TMEM16A) [52, 61]. Hahn et al*.* (2009) suggested that both ANO and TMC proteins share high sequence similarity and the same membrane topology and reported that the regions of the transmembrane domains (TM) of TM1, TM4–TM5 and TM6–TM7, are especially conserved between both families [52]. The protein conformation of ANO1 has been elucidated [62, 63]. Recently, using the structure of ANO1, the predicted three-dimensional structure of mouse TMC1 was reported [64].

Of the 10 mammalian anoctamin families from ANO1 to ANO10, ANO1 and ANO2 have been reported as calcium-activated chloride channels [52, 61, 65]. In mice, ANO1 and ANO2 are expressed in type I or II taste cells, but their specific roles are unknown [66, 67]. TMC4-mediated current is also inhibited by CaCC (inh)-A01, a specific inhibitor of ANO1 and ANO2 [48]. From this information, TMC4 was considered to have a structure common to that of ANO1 at the inhibition site. However, mTMC4 was activated without intracellular Ca^2+^, in contrast to mANO1. The Ca^2+^ binding sites of mANO1 are N646, E650, E698, E701, E730, and E734 [68–71]. However, when comparing the sequence of mTMC4 and that of mANO1, only one residue, the E513 of mTMC4, was conserved as a site corresponding to the E698 of mANO1; the other five residues were not conserved. We believe that this is related to the insensitivity of mTMC4 to intracellular Ca^2+^.

The anoctamin family is well known as an anion channel because of the well-studied ANO1 and ANO2, but it has recently been reported that ANO4, ANO6, and ANO9 are cation channels not anion channels [72–74]. The existence of cation and anion channels in the same family is common to the TMC family, and it is interesting to compare the similarities and differences between the anoctamin and TMC families.

### Phenotype analysis using *Tmc4*-deficient mice

*Tmc4*-deficient mice were produced using the TALEN method. *Tmc4*-deficient mice presented a significantly reduced glossopharyngeal nerve response to 100 mM and 300 mM NaCl (Fig. 1D). The glossopharyngeal nerve’s response to 300 mM KCl also significantly decreased. In contrast, no significant decrease in the chorda tympani nerve responses of *Tmc4*-deficient mice to NaCl and KCl compared to those of WT mice. In addition, a brief access test was performed as a taste behavior experiment in the WT and *Tmc4*-deficient mice. A significant difference was observed in the lick ratio of the NaCl solution to water, between WT and *Tmc4*-deficient mice. These findings suggest that TMC4 is a novel voltage-dependent chloride channel involved in salt taste reception [46].

### Mathematical analysis for the role of TMC4 in salt taste sensation

Using mathematical model of taste cells, we simulated how the TMC4-mediated Cl^−^ current is involved in the generation of action potentials in taste cells induced by salt taste stimuli. The taste cell model was based on the Kimura model [75] and updated according to the experimental data of Ma et al*.* [76] and our experimental data for TMC4. The simulations showed that the model taste cells expressing TMC4 had shortened action potential durations.

**Fig. 2:**
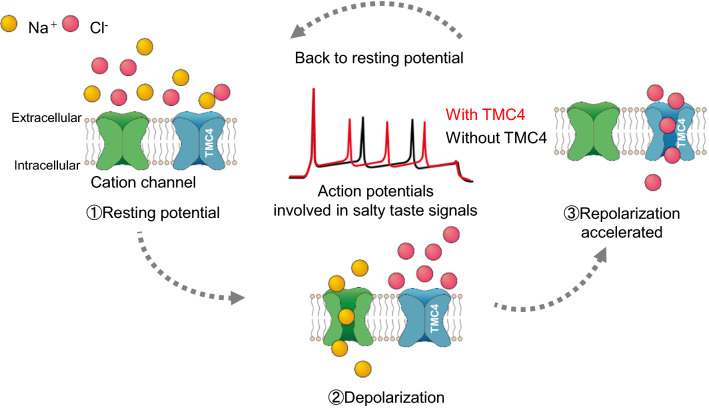
Schematic representation of salt taste reception involving TMC4. The TMC4-expressing taste cell is not activated at the resting potential. Exposure of the taste cells to the stimulus of highly concentrated NaCl induces depolarization via the Na^+^ influx through sodium or cation channels, which triggers an action potential generation for salt taste signals. Furthermore, this depolarization activates TMC4. After depolarization, Cl^−^ fluxes into the taste cells through the TMC4, which helps the taste cells return to the resting potential. Consequently, TMC4 may accelerate the cycle of action potentials for salt taste signals. These processes facilitate neurotransmission via high-concentration salt taste cells, with the result that salt taste reception takes place through the glossopharyngeal nerve

Finally, we present a working hypothesis to explain the role of TMC4 in salt taste reception (Fig. 2). When taste cells are stimulated by salt taste stimuli, they are depolarized by a Na^+^ influx through sodium or cation channels. This depolarization triggers an action potential generation that mediates a salt taste signal and activates TMC4 simultaneously. Extracellular Cl^−^ flows into taste cells through activated TMC4, and the taste cell membrane potential returns to the resting level. The taste cell is then ready to receive the next salt taste stimulus. Consequently, TMC4 may be involved in accelerating the action potential cycles for salt taste signals. In addition, the broad anion selectivity of TMC4 suggested by whole-cell patch-clamp experiments might be an indication that TMC4 is the key molecule in explaining the anion effect.

## Conclusion and perspectives

Following the discovery of TMC4, we have published several reports using hTMC4. Several sensory tests have reported that human salt taste reception is affected by temperature, pH, and non-steroidal anti-inflammatory drugs. We found that hTMC4 was responsive to these stimuli in a manner similar to that of human sensory evaluation [77, 78]. In addition, 3-guanidinyl propanol hydrochloride (3GPrOH), a novel salt taste enhancer, was found in the structure of L-arginine hydrochloride, which is known as a salt taste enhancer [79]. Furthermore, 3GPrOH was reported to significantly enhance hTMC4-mediated currents [79]. Thus, TMC4, an interesting and novel molecule related to salty taste, may be involved in salt taste reception in humans.

As mentioned in the background, in recent years excessive salt intake has been reported to increase the risk of developing a variety of diseases. In 2012, the WHO strongly recommended that the target amount of salt intake for adults be 5 g/day [3]. In response, many countries have considered various approaches to reduce salt intake. Consequently, there have been salt reduction initiatives, such as setting new nutritional guidelines, consumer education on salt, labeling the sodium content on food packages, requesting food companies to reduce salt, and the introduction of the salt tax [80].

The United Kingdom (UK) is one of the leading countries with regard to government interventions to reduce salt intake. Since 2003, the UK has asked food companies to gradually reduce the 40% salt content of processed foods, including bread, which was the main source of salt in the UK [81]. As a result, the UK succeeded in reducing the salt intake amount by 15% in 2011, compared with that before 2001, and reduced the average amount of salt intake from approximately 9.5 g/day to 8.1 g/day [81]. However, in a survey from 2008 to 2019, the daily salt intake did not change, and a further reduction in salt intake was not successful [82]. Even with such strong requests from the government, it will be difficult to meet WHO’s goals. Alonso et al. reported that in 2018, the average salt intake in the UK was approximately 8.4 g/day [83], illustrating that there are still many hurdles to achieve the goals recommended by the WHO. Therefore, the elucidation of salt taste receptors and the development of screening techniques for salt taste enhancers will be powerful tools in realizing this goal.

TMC4 is the first chloride channel reported as a molecule involved in salt taste reception. TMC4 is amiloride insensitive, and is the molecule that could explain the anion effect. It is our expectation that the salt taste enhancer obtained from TMC4 will help achieve the salt reduction target and improve our quality of life.

## Data Availability

Y. Kasahara et al. ‘‘TMC4 is a novel chloride channel involved in high-concentration salt taste sensation.’’ *The Journal of Physiological Sciences* 71.1 (2021):1–14. https://doi.org/10.1186/s12576-021-00807-z. All sequence data used for this study have been deposited in NCBI’s Gene Expression Omnibus (GEO) with the Accession Number GSE175806.

## Abbreviations

ANO: Anoctamin
CvP: Circumvallate papillae
DIDS: 4,4′-Diisothiocyanato-2,2′-stilbenedisulfonic acid
Epi: Surrounding epithelia
FoP: : Foliate papillae
FuP: Fungiform papillae
NMDG: N-methyl-D-glucamine
NPPB: 5-Nitro-2-(3-phenylpropylamino) benzoic acid
RPKM: Reads per kilobase of exon per million mapped reads
TMC4: Transmembrane channel-like 4
WHO: The World Health Organization
3GPrOH: 3-Guanidinyl propanol hydrochloride
